# Supplementary material for: Mitotic clustering of pulverized chromosomes from micronuclei
Source: Nature. 2023 May 10;618(7967):1041–8. doi: 10.1038/s41586-023-05974-0 (PMC10307639; doi:10.1038/s41586-023-05974-0)

nature portfolio

https://doi.org/10.1038/s41586-023-05974-0

Supplementary information

Mitotic clustering of pulverized chromosomes from micronuclei

In the format provided by the authors and unedited

SUPPLEMENTARY INFORMATION GUIDE

Mitotic clustering of pulverized chromosomes from micronuclei

Yu-Fen Lin, Qing Hu, Alice Mazzagatti, Jose Espejo Valle-Inclan, Elizabeth G. Maurais, Rashmi Dahiya, Alison Guyer, Jacob T. Sanders, Justin L. Engel, Giaochau Nguyen, Daniel Bronder, Samuel F. Bakhoum, Isidro Cortés-Ciriano, and Peter Ly

Corresponding authors: peter.ly@utsouthwestern.edu (P.L.)

icortes@ebi.ac.uk (I.C.-C.)

Supplementary Figure 1. Raw, unprocessed images of western blots reported in this study.

Supplementary Table 1. List of balanced chromothripsis events disrupting cancer driver genes in the PCAWG cohort.

Supplementary Table 2. List of oligonucleotide sequences used in this study.

Supplementary Video 1. Live-cell imaging of dCas9-SunTag-labeled Y chromosome mis-segregating into a micronucleus. Y chromosome DYZ1 repeats are labeled with dCas9-SunTag in green; chromatin is labeled with H2B-mCherry in red. Movie shown at 15 min intervals per second.

Supplementary Video 2. Live-cell imaging of dCas9-SunTag-labeled Y chromosome in micronucleis remaining clustered throughout mitosis. Y chromosome DYZ1 repeats are labeled with dCas9-SunTag in green; chromatin is labeled with H2B-mCherry in red. Movie shown at 30 min intervals per second.

Supplementary Video 3. Live-cell imaging of CIP2A-depleted cell exhibiting dispersal of dCas9-SunTag signals upon mitotic entry. Y chromosome DYZ1 repeats are labeled with dCas9-SunTag in green; chromatin is labeled with H2B-mCherry in red. Movie shown at 15 min intervals per second.

Supplementary Video 4. Live-cell imaging of micronucleated, HaloTag-labeled CIP2A puncta remaining clustered throughout mitosis. CIP2A is shown in green; chromatin is labeled with H2B-mCherry in red. Movie shown at 15 min intervals per second.

Source Data for Figures 1-4 and Extended Data Figures 1-9

Supplementary Tables, Supplementary Videos, and Source Data are provided as separate files

Supplementary Figure 1

Extended Data Figure 3a

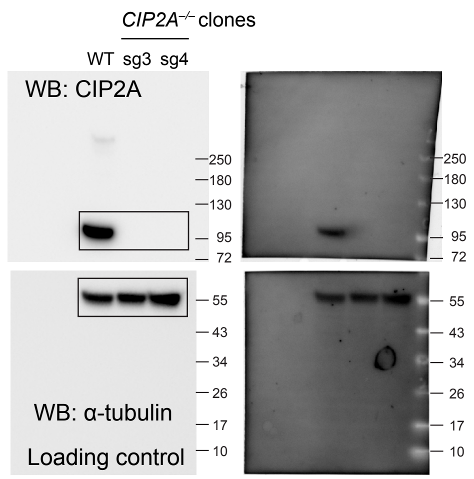

Extended Data Figure 3g

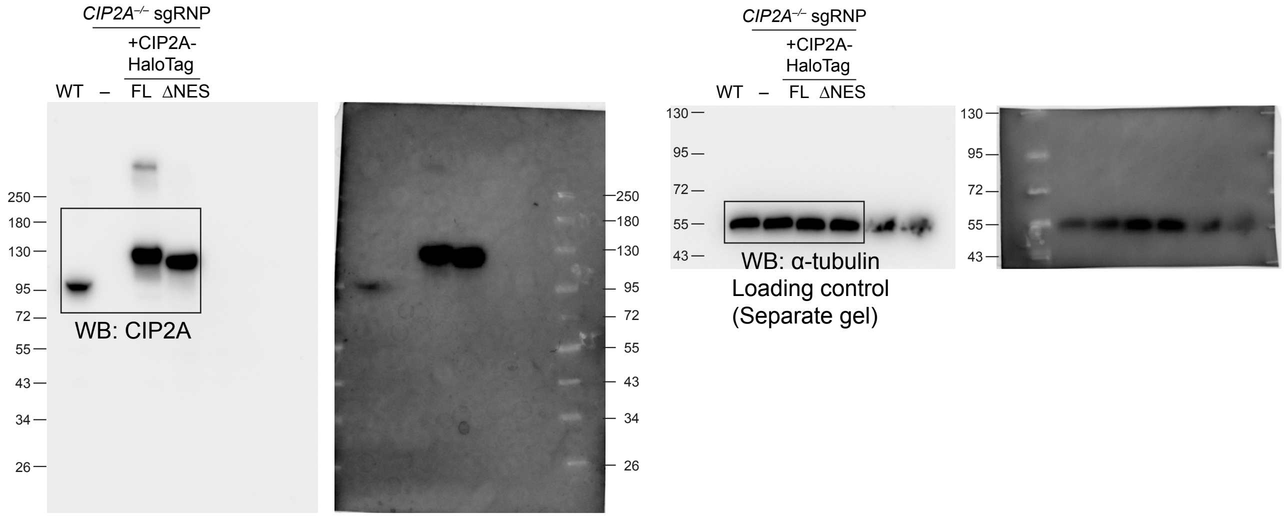

Figure 2f

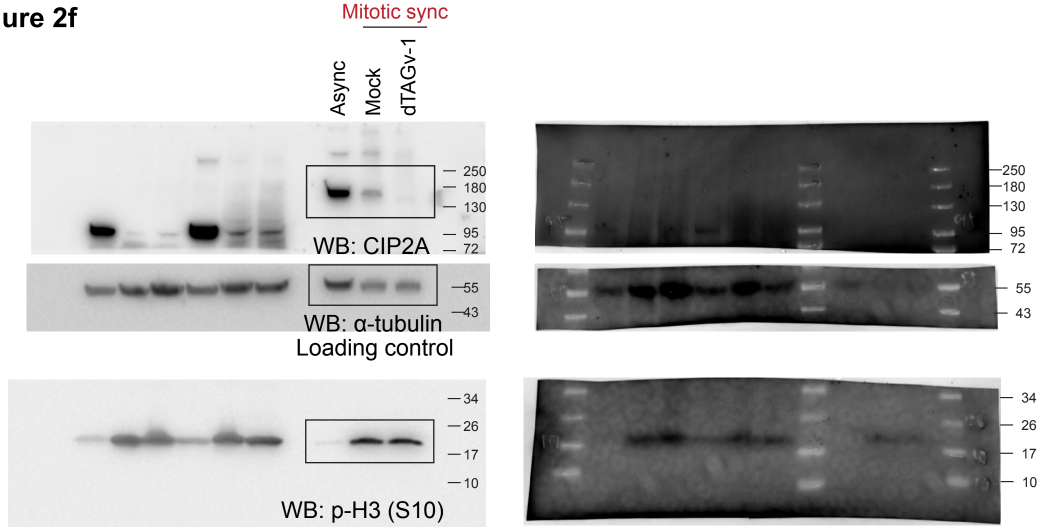

Supplementary Figure 1 (cont.)

Extended Data Figure 4b

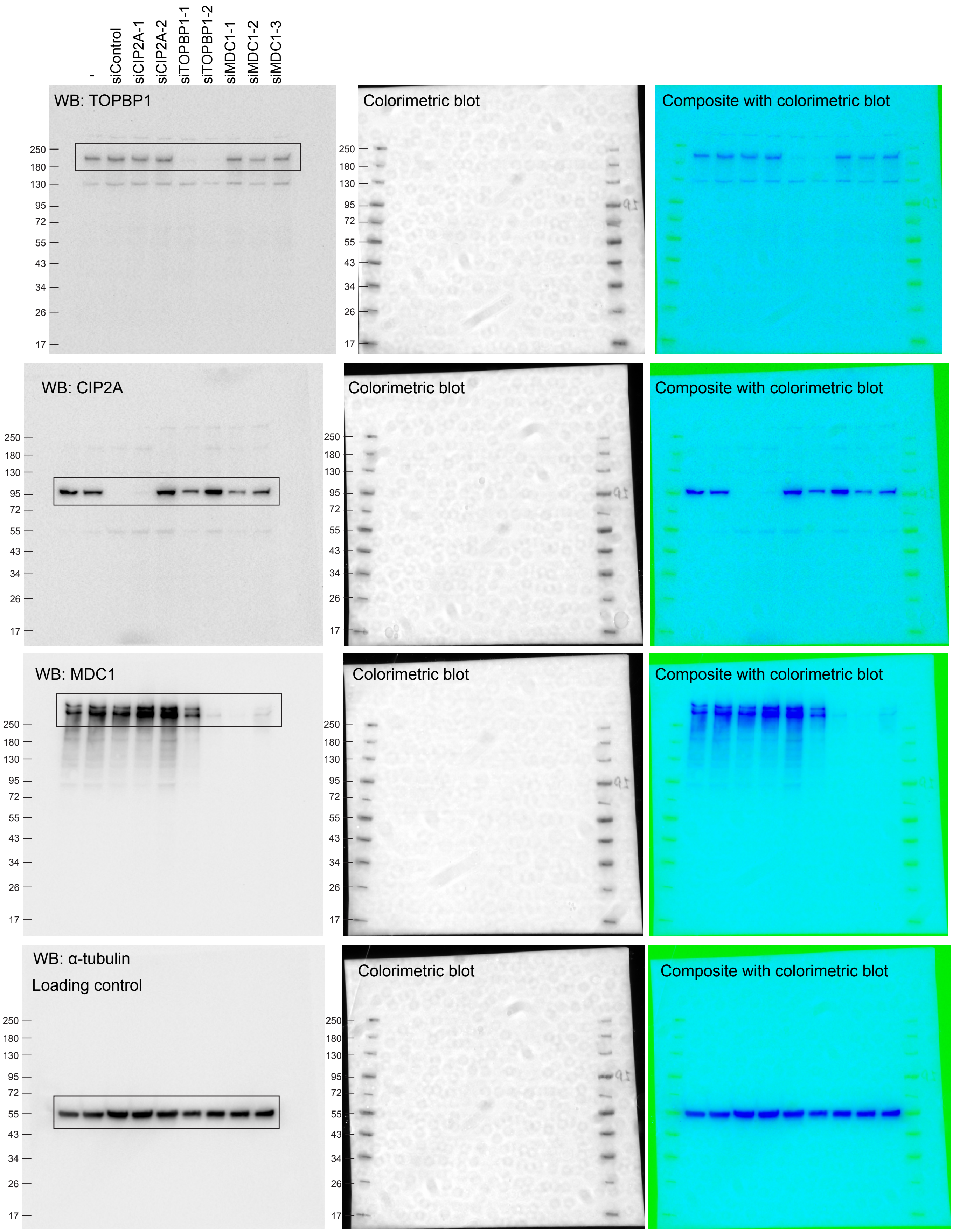

Supplementary Figure 1 (cont.)

Extended Data Figure 5a

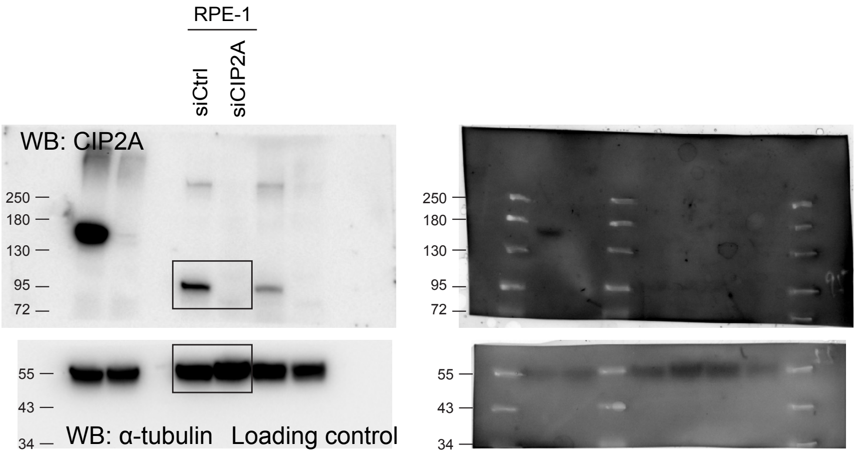

Extended Data Figure 5h

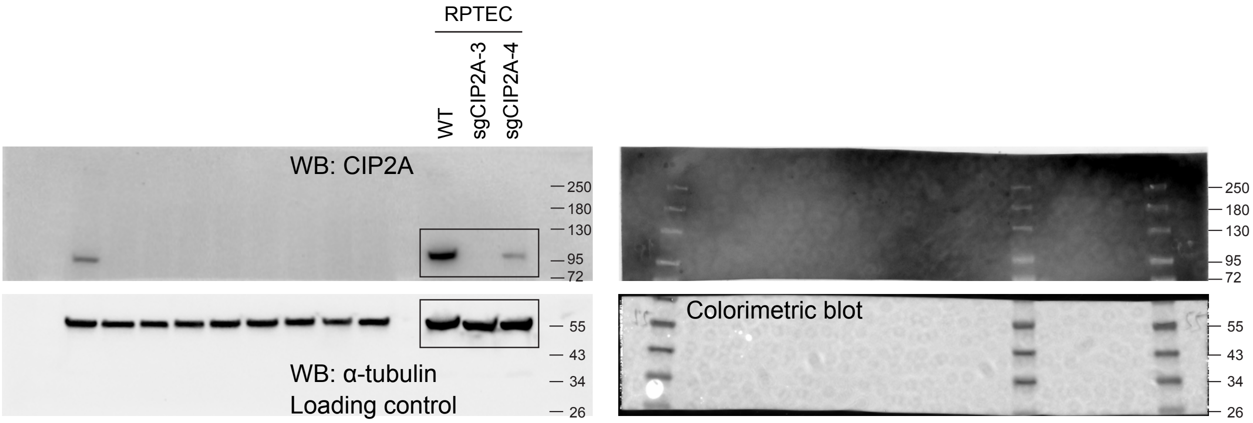

Supplement: Supplementary file 1 — The legends for the Supplementary Information and source data files, and Supplementary Fig. 1. [file 41586_2023_5974_MOESM1_ESM.pdf]
