# Supplementary material for: Mitotic clustering of pulverized chromosomes from micronuclei
Source: Nature. 2023 May 10;618(7967):1041–8. doi: 10.1038/s41586-023-05974-0 (PMC10307639; doi:10.1038/s41586-023-05974-0)
Supplement: Supplementary file 2 — Reporting Summary [file 41586_2023_5974_MOESM2_ESM.pdf]

nature portfolio

Corresponding author(s): Peter Ly, Isidro Cortes-Ciriano

Last updated by author(s): Feb 28, 2023

Reporting Summary

Nature Portfolio wishes to improve the reproducibility of the work that we publish. This form provides structure for consistency and transparency in reporting. For further information on Nature Portfolio policies, see our Editorial Policies and the Editorial Policy Checklist.

Statistics

For all statistical analyses, confirm that the following items are present in the figure legend, table legend, main text, or Methods section.

n/a	Confirmed	
☐	☒	The exact sample size (*n*) for each experimental group/condition, given as a discrete number and unit of measurement
☐	☒	A statement on whether measurements were taken from distinct samples or whether the same sample was measured repeatedly
☐	☒	The statistical test(s) used AND whether they are one- or two-sided<br>*Only common tests should be described solely by name; describe more complex techniques in the Methods section.*
☒	☐	A description of all covariates tested
☐	☒	A description of any assumptions or corrections, such as tests of normality and adjustment for multiple comparisons
☐	☒	A full description of the statistical parameters including central tendency (e.g. means) or other basic estimates (e.g. regression coefficient) AND variation (e.g. standard deviation) or associated estimates of uncertainty (e.g. confidence intervals)
☐	☒	For null hypothesis testing, the test statistic (e.g. *F*, *t*, *r*) with confidence intervals, effect sizes, degrees of freedom and *P* value noted<br>*Give P values as exact values whenever suitable.*
☒	☐	For Bayesian analysis, information on the choice of priors and Markov chain Monte Carlo settings
☒	☐	For hierarchical and complex designs, identification of the appropriate level for tests and full reporting of outcomes
☒	☐	Estimates of effect sizes (e.g. Cohen's *d*, Pearson's *r*), indicating how they were calculated

Our web collection on statistics for biologists contains articles on many of the points above.

Software and code

Policy information about availability of computer code

Data collection	All microscopy images were collected on a DeltaVision Ultra microscope system (GE Healthcare), a Metafer Scanning and Imaging Platform (MetaSystems), or an ImageXpress Confocal HT.ai High-Content Imaging System (Molecular Devices). RNA sequencing was performed on an Illumina NovaSeq 6000 platform by Novogene.<br><br>Software versions: Metafer 4, version 3.13.6, MetaSystems; softWoRx, version 7.2.1, Cytiva
Data analysis	Statistical analyses for cell biological experiments were performed with GraphPad Prism (version 9.5.0) as described in the figure legends. Statistical analyses for RNA sequencing was performed as described in the Methods.<br><br>Software versions: Fiji, version 2.1.0/1.53c; FlowJo, version 10.8.2, BD Biosciences; STAR, version 2.7.4a; HTSeq, version 0.6.1p1; GSEA, version 4.3.2

For manuscripts utilizing custom algorithms or software that are central to the research but not yet described in published literature, software must be made available to editors and reviewers. We strongly encourage code deposition in a community repository (e.g. GitHub). See the Nature Portfolio guidelines for submitting code & software for further information.

Data

Policy information about availability of data

All manuscripts must include a data availability statement. This statement should provide the following information, where applicable:

- Accession codes, unique identifiers, or web links for publicly available datasets
- A description of any restrictions on data availability
- For clinical datasets or third party data, please ensure that the statement adheres to our policy

RNA sequencing data generated by this study are deposited with the European Nucleotide Archive under accession PRJEB59247.

Human research participants

Policy information about studies involving human research participants and Sex and Gender in Research.

Reporting on sex and gender	N/A
Population characteristics	N/A
Recruitment	N/A
Ethics oversight	N/A

Note that full information on the approval of the study protocol must also be provided in the manuscript.

Field-specific reporting

Please select the one below that is the best fit for your research. If you are not sure, read the appropriate sections before making your selection.

☒ Life sciences ☐ Behavioural & social sciences ☐ Ecological, evolutionary & environmental sciences

For a reference copy of the document with all sections, see nature.com/documents/nr-reporting-summary-flat.pdf

Life sciences study design

All studies must disclose on these points even when the disclosure is negative.

Sample size	Sample sizes were not predetermined but were chosen based on current practices in the field. Exact sample sizes and/or the number of independent experiments performed per experiment are indicated in the figure, figure legends, or methods. All experiments reporting P-values were performed independently at least three times.
Data exclusions	No data were excluded from analyses.
Replication	All experiments were independently conducted and reproduced multiple times, as described in the figure legends. All P-values were derived from measurements obtained from experiments conducted independently at least three times. Figures with representative images were reproduced and obtained from at least two or more independent experiments with similar results.
Randomization	N/A
Blinding	Investigators were not blinded during data collection and/or analysis as each series of experiments were performed by an individual researcher.

Reporting for specific materials, systems and methods

We require information from authors about some types of materials, experimental systems and methods used in many studies. Here, indicate whether each material, system or method listed is relevant to your study. If you are not sure if a list item applies to your research, read the appropriate section before selecting a response.

Materials & experimental systems

n/a	Involved in the study
☐	☒ Antibodies
☐	☒ Eukaryotic cell lines
☒	☐ Palaeontology and archaeology
☒	☐ Animals and other organisms
☒	☐ Clinical data
☒	☐ Dual use research of concern

Methods

n/a	Involved in the study
☒	☐ ChIP-seq
☒	☐ Flow cytometry
☒	☐ MRI-based neuroimaging

Antibodies

Antibodies used	For immunofluorescence: 1:500 anti-CIP2A (sc-80659, Santa Cruz), 1:1,000 anti-CIP2A (14805, Cell Signaling), 1:500 anti-TOPBP1 (sc-271043, Santa Cruz), 1:300-500 anti-TOPBP1 (ABE1463, Millipore), 1:1,000 anti-phospho H2AX (S139) (05-636, Millipore), 1:1,000 anti-phospho H2AX (S139) (2577, Cell Signaling), 1:1,000 anti-53BP1 (NB100-304, Novus), 1:1,000 anti-acetyl-histone H3 (Lys 9) (9649, Cell Signaling), 1:1,000 anti-cGAS (15102, Cell Signaling). For immunoblotting: 1:1,000 anti-CIP2A (sc-80659, Santa Cruz), 1:5,000 anti-α-tubulin (3873, Cell Signaling), 1:1,000 anti-TOPBP1 (sc-271043, Santa Cruz), 1:1,000 anti-phospho-histone H3 Ser10 (06-570, Millipore), 1:5,000 anti-MDC1 (ab11171, Abcam). For secondary antibodies, Alexa Fluor-conjugated donkey anti-rabbit and donkey anti-mouse antibodies (Invitrogen) were used for immunofluorescence experiments; horseradish peroxidase-conjugated goat anti-rabbit and donkey anti-mouse antibodies (Invitrogen) were used for immunoblotting.
Validation	The primary antibodies used in this study are commercially available. All critical antibodies were validated by depletion or knockout of the target gene using RNA interference or CRISPR/Cas9 editing, respectively.

Eukaryotic cell lines

Policy information about cell lines and Sex and Gender in Research

Cell line source(s)	DLD-1, human colorectal cancer cells; RPE-1, human retinal pigment epithelial cells; RPTEC, human renal proximal tubule epithelial cells; HeLa, human cervical cancer cells; PC3, human prostate cancer cells; 293T, human embryonic kidney cells; 293GP, human embryonic kidney cells. DLD-1, HeLa, 293T, and 293GP cells were obtained from the cell line repository of Don Cleveland, RPE-1 cells originally generated by Stephen Jackson were obtained through Justin Leung, RPTECs were obtained from Denise Marciano, and PC3 cells were obtained from Sihan Wu.
Authentication	Cell lines were authenticated by morphological characteristics, SNP array analysis, karyotyping, and/or whole-genome DNA sequencing when possible.
Mycoplasma contamination	All cell lines used in this study were routinely confirmed to be free of mycoplasma contamination using the Universal Mycoplasma Detection Kit (ATCC) and by routine DAPI staining.
Commonly misidentified lines (See ICLAC register)	No commonly misidentified cell lines were used in this study
